# The Association between Changes in Coronary Artery Calcium Scores, Dietary Intake, Physical Activity, and Depression Symptoms among the Population of Gaza Strip, Palestine

**DOI:** 10.4314/ejhs.v31i1.11

**Published:** 2021-01

**Authors:** Abdelrazeq Beram, Kate Zinszer, Nouf bamuhair, Samer Abuzerr, Kamal Jabre, Huda Gharbia, Abdel Hamid el Bilbeisi, Awny Ubeid, Waliu Jawula Salisu

**Affiliations:** 1 Prince Naeif Center for Radiodiagnostic, Ministry of Health, Gaza, Palestine; 2 School of Public Health, University of Montreal, Montréal, Canada; 3 University of the Science in the Philadelphia, Pennsylvania, U.S.A.; 4 Advanced Generation International School, Jeddah, Saudi Arabia; 5 Department of Social and Preventive Medicine, University of Montreal, Montréal, Canada; 6 Quality Improvement and Infection Control Unit, Ministry of Health, Gaza, Palestine; 7 Department of Radiology, Al-Shifa Medical Complex, Ministry of Health, Gaza, Palestine; 8 Department of Clinical Nutrition, Al Azhar University of Gaza, Palestine; 9 School of Nursing and Midwifery, Tehran University of Medical Sciences

**Keywords:** Coronary artery calcium score, Depression, Dietary intake, Lifestyle, Strip, Physical activity

## Abstract

**Background:**

This study aimed to identify the association between macronutrient intake, physical activity, and depressive symptoms and changes in coronary artery calcium score among the population of Gaza Strip.

**Method:**

The study sample consisted of 269 individuals who underwent non-enhanced coronary computed tomography using 64-slice MDCT (Siemens, Germany) at Al-Shifa medical complex between September 2017 and January 2018. The study participants were divided into two groups; group one, consisting of coronary calcium calcification scoring (CAC) of greater than zero (CAC score > 0), and group two, CAC less than or equal to zero (CAC score ≤ 0). Data about macronutrient intake, physical activity, and depressive symptoms were collected using a validated self-administered questionnaire. Additionally, the participants' anthropometric characteristics and blood biochemical markers were measured.

**Results:**

Out of 269 participants, 45-recorded CAC score > 0; 72% of them were males with a mean age of 52.6 ± 5.4 years. Significant differences between the two groups in terms of total energy, lipid intake, and carbohydrate were found (P-value of 0.020, 0.012, and 0.034, respectively). No significant differences were recorded in protein intake, physical activity, and depression. Multivariate logistic regression analysis under adjustment for possible confounding factors revealed that macronutrient intake, physical activity, and depressive symptoms were not associated with the development of CAC in two models.

**Conclusion:**

Based on our findings, macronutrient intake, physical activity, and depressive symptoms are not associated with the development of CAC among the population of Gaza.

## Introduction

Ischemic heart disease is a significant cause of death worldwide. The World Health Organization (WHO) has estimated that more than 1 in 7 deaths that occurred in 2015 were caused by ischemic heart disease (1). Coronary artery disease (2) is caused by atherosclerosis of the coronary arteries that leads to a restriction of blood flow to the heart (3). Depending on the degree of stenosis and the characteristics of plaque, patients may experience stable angina or remain asymptomatic until a plaque ruptures and thrombosis occurs, causing acute coronary syndrome (4,5). Multidetector computed tomographic angiography (MDCTA) as a noninvasive examination is a valuable tool for the evaluation of carotid artery disease. It is used to detect early atherosclerotic changes within the carotid arteries, determine stenosis severity, identify the distribution and extent of atherosclerotic plaque, perform plaque characterization, and detect and characterize carotid dissections (6–8).

In recent years, there has been a considerable debate over the dietary intake and lifestyle habits of atherosclerosis. As has been emphasized in several previous studies, many socio-demographic and health-related variables have a direct relationship with developing CAC (9–12). Moreover, dietary intake has shown an association with cardiovascular calcification as high glucose and fat intake improve the plaque buildup inside the arteries (13,14). It is worth mentioning that lifestyle behaviors such as oversleepiness (15,16), lack of physical activity (17–19), and depression (20,21) have been reported as leading causes of CAC. Contrary to this, earlier studies reported that macronutrient intake, physical activity, and depression are not associated with the development of coronary artery calcification (22,23).

Therefore, our study aimed to identify the association between the development of atherosclerosis and macronutrient intake, physical activity, and depressive symptoms among the population of the Gaza Strip.

## Methods

**Context of the study setting**: The study was conducted at Al-Shifa medical complex which is located in the Gaza Strip, a setting suffering prolonged crises and experiencing persistent humanitarian situations. Difficulty accessing care, scarcities in resources, medications, insufficient health facilities, and staff capacity, tremendously challenging living circumstances, a disjointed healthcare system, and snowballing chronic diseases influence the health of people in the Gaza Strip (24–27).

**Study design and population**: The study population consisted of 269 individuals, (both genders, aged ≥ 18 years), who underwent nonenhanced coronary computed tomography between September 2017 and January 2018. Pregnant women, lactating women, and patients with other types of serious illnesses such as cancer or end-stage kidney disease were excluded from the study.

**Coronary Artery Calcification (CAC) measurement**: Coronary artery calcification (CAC) was diagnosed using 64-slice MDCT (Siemens, Germany) at Al-Shifa medical complex. The facility, at the time of this study, was one of few health centers that provided non-enhanced coronary computed tomography in the Gaza Strip. Standard scanning protocol using 2.5-mm thickness without overlap and gap, 400 ms rotation time, 120 kV tube voltage, and 124 mAs (310 mA * 0.4 s) tube current under ECG-gated dose modulation was followed. Coronary artery calcification was defined as more than three contiguous pixels above a CT density of 130 Hounsfield Units. The quantification of coronary artery calcium using ultrafast computed tomography was determined according to Agatston's method (28). The study participants were categorized into two groups; group one with CAC score > 0 and group two with CAC score ≤ 0.

**Assessment of anthropometric measurements**: Height was measured in all patients with a measuring rod attached to the balanced beam scale; the height was reported to the nearest 0.5 cm. Weight (kg) was measured using a standard scale (Seca); the scale was placed on a hard-floor surface; patients were asked to remove their heavy outer garments, and weight was measured and recorded to the nearest 0.1 kg. The Body Mass Index (BMI) was calculated by dividing weight in kilograms by the square of height in meters.

**Blood biochemical markers**: Blood samples were collected from the antecubital vein of each participant after 12 hours of fasting by registered nurses. Samples were directly sent to a licensed laboratory after collection in a Serum Separating Tube (SST). The extracted serum was analyzed for high-density lipoprotein cholesterol (HDL-c) mg/dl, low-density lipoprotein cholesterol (LDL-c) mg/dl, triglyceride (TGs) mg/dl, fasting serum glucose (mg/dL), total cholesterol (mg/dL), alanine aminotransferase (ALT), and gamma-glutamyl transferase (GGT). Additionally, LDL-c was calculated using the Friedwald formula; Mindray BS-300 chemistry analyzer instrument was used for blood chemistry analysis (29).

**Dietary intake assessment**: Data about dietary intake were collected by an expert nutritionist, using a validated semi-quantitative food frequency questionnaire (FFQ). The FFQ is relatively easy and inexpensive to administer and can be used to measure dietary intake over a prolonged period (29). The FFQ in our study contains a list of 98 food items; it was developed and validated among the Palestinian population in 2014 (30). All participants were asked to estimate the number of times per day, week, or month he/she consumed these particular food products and the amount usually eaten per food item by making comparisons with the specified reference portion. Everyday household items, including measuring cups, spoons, and a ruler, were shown to assist the participants in the estimation process. The answer categories ranged from 1 to 7 times (7 categories) including never, one to three times per month, one to two times per week, three to four times per week, five to six times per week, one time per day, or two to three times per day. The food composition of mixed dishes was determined by using traditional recipes consumed in the country. The mean intake of each food item in grams was calculated by multiplying the specified portion size by the average reported frequency. In addition, the USDA food composition tables were used to analyze nutrients consumption. Then, the participants were classified into four quartiles based on macronutrient intake (Q1 ∼ Q4).

**Physical activity**: Data on physical activity were obtained using the International Physical Activity Questionnaire (IPAQ short version) (31). The internationally accepted protocol was used to estimate the weekly calorie expenditure expressed as metabolic equivalents per week (MET/wk). The IPAQ scoring protocol assigns the following MET values to walk, moderate, and vigorous-intensity activity: 3.3 METs, 4.0 METs, and 8.0 METs, respectively (32).

**Covariates assessment**: A self-administered questionnaire was used to collect data about socioeconomic and demographic characteristics, past medical history, medication use, and health-related information of the study participants. A pilot study was carried out on twenty participants to enable the researchers to examine the tools of the study. The questionnaire and data collection process were modified according to the result of the pilot study.

**Depression assessment**: Depression was evaluated by the Center for Epidemiological Studies Depression Scale (CESD), which is a popular assessment tool that has broad applicability in the general population (33).

**Blood pressure assessment (BP)**: The average of triplicate BP measurements was recorded using a stethoscope and mercury sphygmomanometer from the left arm (mmHg). The participants were seated for fifteen minutes before the measurement in a quiet room without having any food, beverage, or smoke. The world health organizations' (WHO's) definition of high BP as a systolic BP equal to or above 140 mmHg and diastolic BP equal to or above 90 mmHg was adopted (34).

**Ethical issues**: The study was approved by the General Directorate of Human Resources Development in the Palestinian Ministry of Health. Moreover, written consent was obtained from each participant.

**Statistical analyses**: All statistical analyses were performed using statistical package for social science (SPSS) version 20. Normally distributed variables were presented as the mean ± SD, and skewed variables were presented as the median (interquartile range). Continuous variables were compared using mindependent t-test between groups one and two (CAC score > 0 and CAC score ≤ 0). The multivariate logistic regression model was employed to identify Hazard Ratios (HRs) for the development of CAC with 95% confidence intervals (CIs) across the quartile of macronutrient intake, physical activity, and depression after adjustment of possible confounding factors. The lowest quartile group was considered as a reference. Two models were studied; the first model was adjusted for age and sex, and the second model for smoking, physical activity, glucose, TGs, HDL-c, LDL-c, BMI, and hypertension, which were considered statistically significant in the univariate logistic regression (*P* values <0.05).

## Results

Among 269 participants, 45 individuals had a CAC score > 0. And, 72% of them were males with a mean age of 52.6 ± 5.4 years. Statistically significant associations were found between groups one and two. The individuals of group one were of older ages. They had higher BMI, systolic and diastolic BP, fasting serum glucose, lipid profiles levels, diabetes, hypertension, GGT, ALT, lower HDL-c and total energy (*p* values < 0.001). On the other hand, there were no statistically significant relationships between the two groups concerning smoking status, fat intake, carbohydrate intake, protein intake, physical activity, and depression status (*p* values > 0.05) (Table 1).

**Table 1:** Characteristics of the study participants according to coronary artery calcification

Variable	CAC score		*P*-value

	CAC score ≤ 0	CAC score > 0	
Frequency (%)	224 (83.27)	45 (16.73)	-
Male	172 (77)	32 (72)	0.001
Age (years)^a^	47.5 (5.9)	52.6 (5.4)	0.001
Smoking (%)	81 (36)	17 (38)	0.367
Body mass index (kg/m^2^)^a^	26.2 (3.1)	27.5 (3.2)	0.001
Systolic blood pressure (mmHg)^a^	119.5 (12.4)	122.4 (12)	0.001
Diastolic blood pressure (mmHg)^a^	76.3 (9.6)	78.9 (9.7)	0.001
Fasting serum glucose (mg/dl)^a^	98.1 (15.2)	104.1 (22.1)	0.001
Total cholesterol (mg/dl)^a^	206.9 (36.5)	218.8 (38.1)	0.001
Low-density lipoprotein-cholesterol (mg/dl)^a^	130.3 (32.9)	141.5 (34.4)	0.001
Triglycerides (mg/dl)^a^	135 (143.3)	161 (168.5)	0.001
High-density lipoprotein-cholesterol (mg/dl)^a^	51.5 (12.3)	49.1 (11.0)	0.001
Diabetes (%)	20 (9)	9 (19)	0.001
Hypertension (%)	43 (19)	13 (29)	0.001
Gamma glutamyl transferase (U/L)^b^	36 (24–55)	41 (28–67)	0.001
Alanine aminotransferase (U/L)^b^	26 (19–38)	30 (22–41)	0.001
Total energy (kcal per day)^b^	1544.1(1162.0–1820.0)	1491.8(6484.2–1662.7)	0.003
Fat (g per day)^b^	17.1 (13.1–21.2)	15.6 (13.4–22.5)	0.431
Carbohydrate (g per day)^b^	73.9 (68.0–79.2)	78.2 (69.1–83.4)	0.521
Protein (g per day)^b^	14.2 (11.9–16.1)	14.4 (13.2–16.3)	0.997
Physical activity (MET/week)^b^	997.7 (447–1923)	1162.8 (545–2121)	0.749
Depression (CES-D score)^b^	5 (1–10)	5.5 (1–11)	0.495

Abbreviations: (a) mean (standard deviation), (b) median (interquartile range), (%) percentage, (kg/m2) Kilogram per square meter, (mmHg) millimeter of mercury, (mg/dl) Milligrams per deciliter, (U/L) Units per liter, (MET) metabolic equivalent, and (CES-D) Center for Epidemiologic Studies Depression Scale

**Figure 1:**
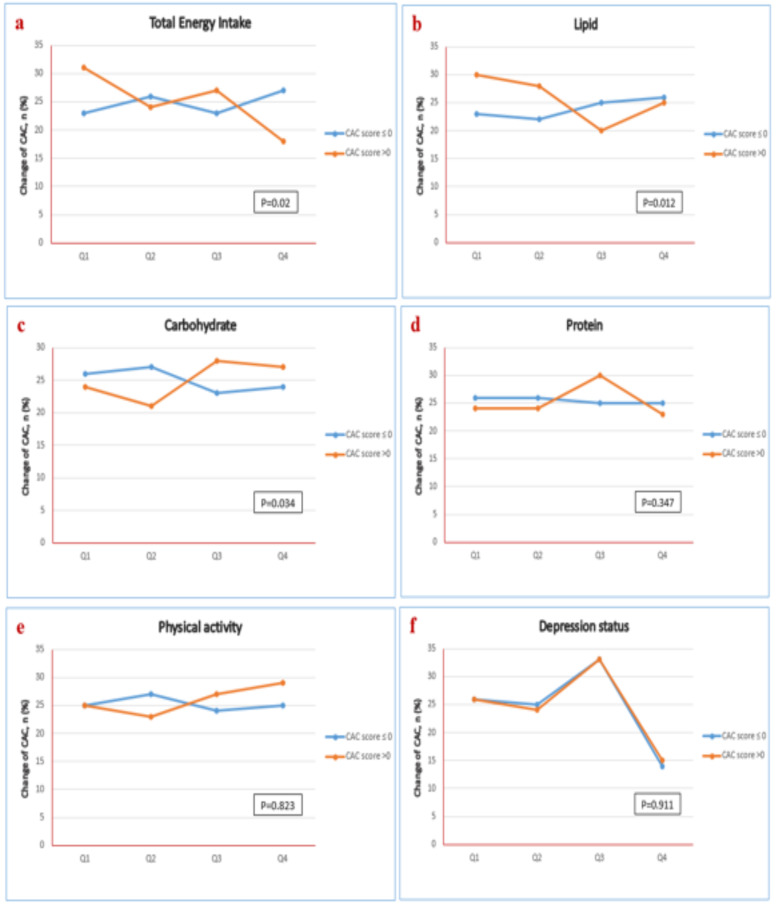
Correlation between CAC change and a) Total energy intake, b) Lipid intake, c) Carbohydrate intake, d) Protein intake, e) Physical activity, f) Depression status

The groups were compared based on quartiles of total energy intake, macronutrient intake, physical activity, and depression status. The results showed a statistically significant association with total energy, lipid, and carbohydrate intake (*p* values = 0.02, 0.01, 0.03, respectively). Nevertheless, no statistically significant associations were found for protein intake, physical activity, and depression status (*p* values > 0.05) (Figure 1).

The multivariate logistic regression analysis was employed to assess the association between group one and quartile categories of macronutrient intake, physical activity, and depression. Two models were examined. The first model was adjusted for age and sex, whereas the second model was adjusted for smoking, physical activity, glucose, TGs, HDL-c, LDL-c, BMI, and hypertension. Even though several variables were significantly associated with CAC progression in univariate analyses, they all lost their significance in multivariable analysis while adjusting for potential confounders (p values for trends > 0.05) (Table 2).

**Table 2:** Multivariate logistic regression models for CAC score based on quartiles of macronutrients intake, physical activity, and depression

Variable/CAC change > 0	Q1	Q2	Q3	Q4	*P*-value for trend
**Total energy**					
N (total /CAC change > 0)	68/12	67/14	67/10	67/9	
Model one	1.00	0.780 (.172–.486)	0.377 (.062–.950)	0.754 (0.383 .544)	0.346
Model two	1.00	0.822 (.350–.560)	0.097 (.823–1.340)	0.874 (0.612–.487)	0.422
**Carbohydrate %**					
N (total /CAC change > 0)	68/13	67/12	67/10	67/10	
Model one	1.00	0.780(.857–1.041)	0.648 (.341–.254)	0.253 (0.256 .580)	0.611
Model two	1.00	.522 (.238–.760)	0.855 (.716–1.245)	0.654 (.711–2.051)	0.247
**Protein%**					
N (total /CAC change > 0)	68/10	67/16	67/9	67/10	
Model one	1.00	0.258 (.631–1.166)	0.875 (.836–1.225)	0.658 (.813–1.021)	0.645
Model two	1.00	0.970 (0.741–.950)	0.395 (.658–1.324)	0.270 (0.421–.660)	0.983
**Fat%**					
N (total /CAC change > 0)	68/12	67/11	67/8	67/14	
Model one	1.00	0.142 (.022–.366)	0.342 (.442–.842)	0.457 (.298–.870)	0.163
Model two	1.00	0.882 (.734–1.110)	0.748 (.569–.732)	0.745 (.665–.892)	0.146
**Physical activity**					
N (total /CAC change > 0)	67/9	67/13	67/13	67/10	
Model one	1.00	0.774 (.258–1.311)	0.474 (.860–.899)	0.621 (.426–.897)	0.578
Model two	1.00	0.447 (.232–1.241)	0.841 (.451–.660)	0.832 (.857–.938)	0.135
**Depression**					
N (total /CAC change > 0)	68/12	67/12	67/10	67/11	
Model one	1.00	0.763 (.678–.844)	0.216 (.523–.364)	0.680 (.679–.356)	0.228
Model two	1.00	0.543 (.567–.855)	0.652 (.413–.440	0.450 (.699–.930)	0.238

## Discussion

Our study showed that total energy intake, carbohydrate intake, protein intake, fat intake, physical activity, and depressive symptoms were not associated with the development of CAC after adjustment of possible confounding factors in the Gaza Strip.

Although recent studies have reported that excessive fat intake may accelerate the development of coronary vascular disease (CVD) (14), our findings were more compatible with the other earlier studies which suggest that dietary pattern and macronutrient intake may not be the leading causes of the atherosclerosis disease and carotid artery calcification after the adjustment for various confounders (22,23,35).

Even though the BMI level among group one (CAC scores > 0) was higher than group two (CAC score ≤ 0), to no small extent, it can account for the weight loss due to the higher activity among the individuals of the second group. However, no significant relationship was achieved between the development of atherosclerosis and physical activity in this study. In contrast, many studies have indicated the importance of physical exercises in reducing the risk of atherosclerosis (18,32,37).

Besides, an inverse relationship was found between the physical activity and incident of CAC. This was demonstrated in a multi-ethnic study (17).

Our results showed no association between the development of cardiovascular disease (CVD) and depression status. On the contrary, depressive symptoms have been consistently associated with a higher risk of coronary heart disease (CHD) (20,21,38). Our result in this regard could be attributed to the variation in the sociocultural characteristics among the societies. As well, women seem to be more strongly affected by psychosocial stressors related to CVD and depression, and by direct/indirect effects of chronic stress compared to men (39). This seems to reflect in our findings as about 32 male participants had a CAC score > 0.

Large-scale research in the future is recommended to confirm our results and to overcome the current shortcomings of this study, which are related to the small size of the study sample. Two CT scan machines that will provide non-enhanced coronary computed tomography are expected to be operational in the Gaza Strip soon. This will improve service delivery in the area and enable researchers to have access to more significant prospective participants for future studies.

In conclusion, our current findings indicate that the levels of macronutrient intake, physical activity, and depressive symptoms are not associated with CAC scores among the Palestinian population of the Gaza Strip. However, we recommend further research on a larger-scale in this area to confirm our findings.
